# Diatom Derived Polyunsaturated Aldehydes Do Not Structure the Planktonic Microbial Community in a Mesocosm Study

**DOI:** 10.3390/md10040775

**Published:** 2012-03-28

**Authors:** Carsten Paul, Anna Reunamo, Elin Lindehoff, Johanna Bergkvist, Michaela A. Mausz, Henrik Larsson, Hannes Richter, Sten-Åke Wängberg, Piia Leskinen, Ulf Båmstedt, Georg Pohnert

**Affiliations:** 1 Department for Bioorganic Analytics, Friedrich Schiller University Jena, 07743 Jena, Germany; Email: carsten.paul@JSMC.info (C.P.); michaela.mausz@uni-jena.de (M.A.M.); hannes.richter@uni-jena.de (H.R.); 2 Department of Biology, Division of Genetics and Physiology, University of Turku, FI-20014 Turku, Finland; Email: anna.lindroos@utu.fi (A.R.); piiles@utu.fi (P.L.); 3 Umeå Marine Sciences Centre, Umeå University, 91020 Hörnefors, Sweden; Email: elin.lindehoff@emg.umu.se (E.L.); henrik.larsson@umf.umu.se (H.L.); ulf.bamstedt@emg.umu.se (U.B.); 4 Department of Ecology and Environmental Sciences, Umeå University, 901 87 Umeå, Sweden; 5 Department of Biological and Environmental Sciences, University of Gothenburg, SE-405 30 Göteborg, Sweden; Email: johanna.bergkvist@dpes.gu.se (J.B.); sten-ake.wangberg@dpes.gu.se (S.-A.W.); 6 Leibniz Institute for Natural Product Research and Infection Biology, Hans Knöll Institute (HKI), 07745 Jena, Germany

**Keywords:** mesocosm, plankton interactions, aldehydes, oxylipins

## Abstract

Several marine and freshwater diatoms produce polyunsaturated aldehydes (PUA) in wound-activated processes. These metabolites are also released by intact diatom cells during algal blooms. Due to their activity in laboratory experiments, PUA are considered as potential mediators of diatom-bacteria interactions. Here, we tested the hypothesis that PUA mediate such processes in a close-to-field mesocosm experiment. Natural plankton communities enriched with *Skeletonema marinoi* strains that differ in their PUA production, a plankton control, and a plankton control supplemented with PUA at natural and elevated concentrations were observed. We monitored bacterial and viral abundance as well as bacterial community composition and did not observe any influence of PUA on these parameters even at elevated concentrations. We rather detected an alternation of the bacterial diversity over time and differences between the two *S. marinoi *strains, indicating unique dynamic bacterial communities in these algal blooms. These results suggest that factors other than PUA are of significance for interactions between diatoms and bacteria.

## 1. Introduction

Diatoms comprise a group of phototrophic microalgae with worldwide dominance in higher latitudes. Some diatom species and isolates are known to produce a multitude of fatty acid-derived secondary metabolites such as polyunsaturated aldehydes (PUA) [1]. A negative effect of PUA on the reproduction success of herbivorous copepods was proposed by several authors based on evidence from feeding experiments [2], application of pure PUA [3] or PUA encapsulating liposomes [4] (reviewed in [5,6]). While some evidence for the action of PUA was found in the field [7], several investigations failed to correlate diatom abundance with reproduction failure of copepods [8,9] as well as PUA production with lower egg hatching success [10,11,12,13]. Additionally, modeling studies suggest that PUA-based chemical defense against herbivory is not advantageous for a diatom population, since it cannot be considered as an evolutionary driving force [14]. Several studies point to other potential functions of this class of metabolites such as infochemicals involved in diatom-diatom interactions [15,16] or mediators of the microbial community around diatom cells [17]. For such interactions PUA excretion by intact diatoms would be required and indeed, PUA release (hepta- and octadienal) was recently shown for the species *Skeletonema marinoi* in culture [16] as well as in mesocosm experiments [18]. PUA were also detected in subnanomolar concentrations during a *S. marinoi* bloom in the Adriatic Sea [19] indicating that e.g., co-existing bacteria are exposed to these metabolites during a diatom bloom. Several adverse effects of PUA on microorganisms have been reported and it has been suggested that diatom-derived PUA can regulate the bacterioplankton community [3,17,20]. This assumption is supported by the observation that PUA are generally strongly bioactive, and capable of disturbing normal cell functions in several organisms [21]. However, the results obtained from laboratory studies so far lacked experimental verification under ecologically relevant conditions. While agar diffusion assays were used to assess the cell toxicity of PUA on bacteria isolated from habitats unrelated to diatom distribution [3], Ribalet *et al.* tested, among others, bacterial strains isolated from a *S. marinoi* bloom in liquid nutrient enriched bacterial growth medium. Concentrations used in these experiments were in the high micromolar range [17] exceeding average natural conditions in the water by several orders of magnitude [18,19]. Most ecologically relevant is a recent study that applied 7.5 nM PUA, which is in the range of natural concentrations, to bacterial strains isolated from the Mediterranean Sea, and observed group specific effects [20]. However, all these experiments are laboratory tests based on application of pure PUA on cultivable bacterial strains.

Given the evidence of an activity of PUA on bacteria and the notion that these metabolites are indeed found in the seawater, we aimed to investigate their role in a set-up that is close to a field situation. We therefore designed a mesocosm experiment to test the hypotheses that (1) PUA influence the abundance of the microbial community, including bacteria and viruses, and (2) PUA produced by diatoms influence the biodiversity of the bacterial plankton communities. As a test organism, we used the diatom *S. marinoi*, since its PUA content and the release of these metabolites into seawater has been documented in laboratory, mesocosm and field experiments [16,18,19]. Despite significant quantitative variability between isolates, it can be stated that this species generally produces the shorter chain length PUA, 2,4-heptadienal, 2,4-octadienal, and sometimes to a minor extent 2,4,7-octatrienal, while it lacks C10 PUA. *Skeletonema* spp. are regularly found at the study site even if they contribute often only little to the phytoplankton community and are thus ideally suited to manipulate existing communities. The experimental set-up was rigorously replicated and included mesocosms where cultivated PUA-producing *S. marinoi *strains were used to trigger a diatom bloom and mesocosms where synthetic PUA were introduced in different concentrations as well as controls. We undertook a comprehensive monitoring of phytoplankton development, chemical exudates, bacterial abundance and community composition as well as viral abundance and observed no effects by PUA-producing diatoms. Additions of synthetic PUA in natural and above natural (*ca.* 1000×) concentrations did not result in major changes of the bacterial and viral abundance.

## 2. Results

### 2.1. Phytoplankton Development

**Figure 1:**
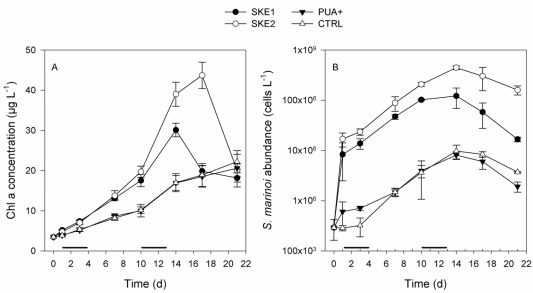
Development of (**A**) total chlorophyll a concentration and (**B**) the cell abundance of *Skeletonema marinoi* during the time course of the experiment. SKE 1 and SKE2 were inoculated with two different *S. marinoi *strains at day 0. The black lines on top of the time axis indicate the first (low PUA concentration) and second (high PUA concentration) PUA addition to the PUA+ treatment.

In the following, the treatments are abbreviated with SKE1 and SKE2 for the mesocosms inoculated with the two *S. marinoi *strains, PUA+ for the mesocosms with additions of PUA and CTRL for the untreated control. All treatments started with a total chlorophyll a concentration of 3.5 µg·L^−1^. A phytoplankton bloom developed in SKE1 and SKE2 as indicated by an increasing chlorophyll a concentration. SKE2 reached the highest chlorophyll a concentration of more than 40 µg·L^−1^, peaking at day 17. In SKE1, chlorophyll a was peaking at day 14 with a maximal concentration of 30 µg·L^−1 ^(Figure 1A). These two treatments showed significant differences to each other at day 14 and day 17 (*P* < 0.001).

PUA+ and CTRL showed very similar progressions in their chlorophyll a concentration and no significant differences were recorded at any time point (*P *> 0.66 for all comparisons). In these treatments, chlorophyll a concentration steadily increased and from day 7 to day 14; chlorophyll a values were significantly lower compared to SKE1 and SKE2 (*P *< 0.02 for all comparisons). *S. marinoi* cell counts revealed that in CTRL and PUA+ treatments a population of *S. marinoi* developed, which was consistently lower in abundance compared to the SKE1 and SKE2 treatments (Figure 1B). *S. marinoi* cell counts in PUA+ showed no significant difference to CTRL over the course of the experiment (*P *= 1 for all comparisons). SKE2 showed a significantly increased *S. marinoi *cell abundance in comparison to SKE1 at day 10 and day 17 (*P *= 0.001 and *P *< 0.001 for day 10 and day 17, respectively).

**Figure 2:**
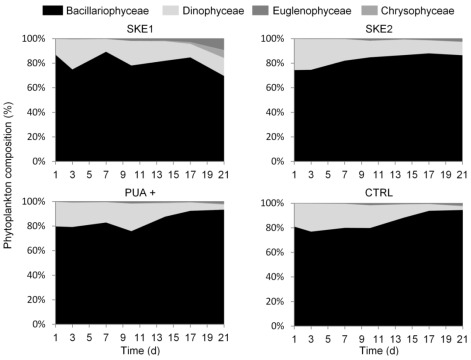
Development of phytoplankton composition in SKE1 and SKE2, PUA+ and CTRL. Only phytoplankton classes that contribute more than 0.4% to the total biomass are displayed. PUA additions to the PUA+ were between days 1 and 4 (low concentration) and between days 10 and 13 (high concentration).

The phytoplankton composition was dominated in every treatment by diatoms reaching maximum 94% and minimum 70% of total biomass at the end of the experiment (day 21) (Figure 2). *S. marinoi* accounted for up to 20% of the biomass in SKE1 and up to 40% of the biomass in SKE2 (data not shown). The second most abundant class of algae was dinoflagellates. These accounted for approximately 4% of the total biomass at day 21 in PUA+ and CTRL and 14% and 11% in SKE1 and SKE2, respectively. Euglenophyceae and Chrysophyceae contributed less to the biomass and were most abundant in SKE1 with respectively 6% and 9% at the end of the experiment. In all other treatments, these classes were only present in very minor amounts.

### 2.2. Bacterial and Viral Abundance

**Figure 3:**
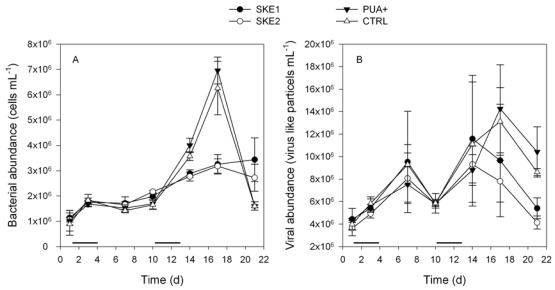
Bacterial (**A**) and viral (**B**) abundance during the experiment. The black lines on top of the time axis indicate the two periods of PUA addition.

The initial bacterial abundance was very similar in all treatments with roughly 10^6^ cells·mL^−1^ at day 1, and showed no significant difference between the four treatments (*P *> 0.829). Irrespective of the first PUA addition or the developing *S. marinoi* blooms, the bacterial abundance developed uniformly in all treatments until day 10 when densities of ca. 1.8 × 10^6^ cells·mL^−1^ were reached, again not showing any significant differences between any treatments (*P *> 0.954 for all comparisons) (Figure 3A). At day 17 PUA+ and CTRL showed significantly higher bacterial abundances compared to SKE1 and SKE2 (*P *< 0.001 for all 4 comparisons). Bacterial abundance in SKE1 and SKE2 did not differ significantly during the entire experiment (*P *> 0.088 at any time point, Figure 3A).

Initial viral abundance in all treatments was at 4 × 10^6^ particles·mL^−1^ and increased to a maximum of 14 × 10^6^ particles·mL^−1^ in PUA+ by day 17. No significant differences were observed until day 14 (*P *> 0.416). After day 14 PUA+ contained significantly more virus like particles than SKE1 and SKE2 (*P *< 0.05 for all comparisons). CTRL and PUA+ as well as SKE1 and SKE2 did not differ in their virus abundance at any time point (*P *> 0.572 at each time point, Figure 3B).

### 2.3. Dissolved PUA Concentration

**Figure 4:**
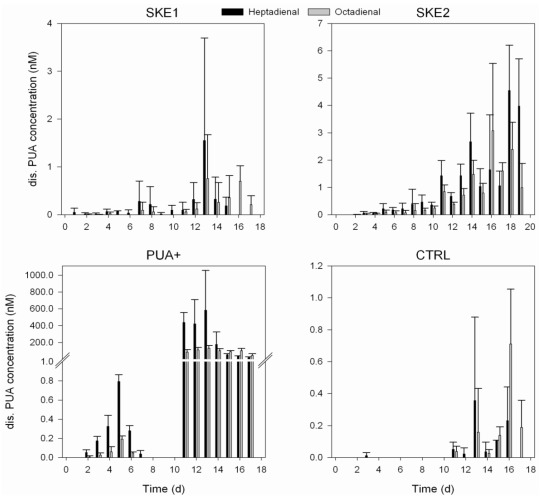
Daily concentration of dissolved PUA in all treatments. Please note the different scales on y-axes in each graph. SKE2 has additionally a different time axis due to a prolonged algal growth in this treatment.

To mimic natural concentrations occurring during diatom blooms, the first PUA addition was adjusted to result in subnanomolar concentrations of hepta- and octadienal in the mesocosms [19]. PUA were added after the sampling at days 1, 2, 3 and 4. A second repeated addition with 1000fold more PUA was conducted at days 10, 11, 12 and 13. The concentration of dissolved PUA found in the PUA+ treatment reflected the PUA addition. From day 2 to day 7, we detected PUA starting from 0.05 nM for heptadienal and very minor amounts of octadienal. At day 2 the concentrations were increasing to a maximum of 0.8 nM heptadienal and 0.2 nM octadienal at day 5. From day 5 to day 7 the measured PUA concentration decreased again until it was not detectable from day 8 to day 10, which marks the start of the second PUA addition period (Figure 4). After the repeated addition of high PUA concentrations up to 570 nM heptadienal and 125 nM octadienal were reached at days 11 to 13. Later, the heptadienal concentration decreased. In the CTRL treatment, neither heptadienal nor octadienal could be detected during the first 10 days of the experiment, excluding a very low amount of heptadienal detected at day 3. After day 10, heptadienal as well as octadienal in variable but low amounts could be detected in the CTRL mesocosm but not exceeding concentrations of 0.8 nM (Figure 4). 

In SKE1 subnanomolar concentrations of dissolved PUA were detected during the beginning of the experiment and only towards the end (day 13) were levels around 1 nM or higher observed. In general, PUA levels were higher in SKE2. Similar to SKE1 only subnanomolar concentrations of hepta- and octadienal were detected during the first 10 days. Towards the end of the experiment PUA levels in SKE2 reached up to 4 nM heptadienal (day 18) and up to 3 nM octadienal (day 16, Figure 4).

### 2.4. Bacterial Community Composition

**Figure 5:**
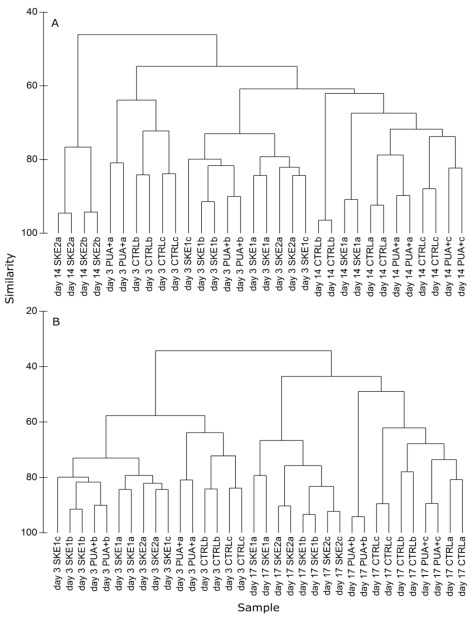
Cluster analysis of T-RFLP samples taken during low (day 3), directly after high (day 14) PUA addition period, and towards the end of the experiment (day 17). Cluster analysis of the day 3 and day 14 samples (**A**), in comparison to a cluster analysis of day 3 and day 17 (**B**). Replicates that did not fulfill our reproducibility criteria were discarded from the analysis. Each sample was measured twice and both technical replicates were used for the analysis. Technical replicates obtained from the same biological sample are indicated with the same letter.

The cluster analysis (Figure 5) included samples taken during the first PUA addition (day 3), samples taken directly after the second PUA addition period (day 14) and samples taken towards the end of the experiment. On day 3, CTRL and PUA+ did not group differently from each other, indicating a homogeneous bacterial community in both treatments. Additionally, neither SKE treatments differed from the control and neither differed from each other. In addition, at day 14 we could not discriminate between CTRL and PUA+. All samples, with the exception of SKE2, grouped together (Figure 5A). This unique bacterial community in SKE2 was not observable at day 17 (Figure 5B). In contrast to day 14, at day 17 SKE1 and SKE2 grouped closely together, but clustered apart from CTRL and PUA+. In total, all samples of day 3 grouped separately from samples of day 17, indicating a change of the bacterial community over time (Figure 5B).

## 3. Discussion

Addition of nutrients and inoculation of the diatom *S. marinoi* allowed us to generate diatom dominated phytoplankton blooms in the mesocosms SKE1 and SKE2. We reached a total chlorophyll a concentration of up to 40 µg·L^−1^ for SKE2 and 30 µg L^−1^ for SKE1. The chlorophyll a concentration was thus 6 to 8 fold higher than typically observed during a spring bloom in the Bothnian Sea which reaches around 5 µg·L^−1^ [22]. Chlorophyll a concentrations were comparable to those typically obtained in fertilized diatom mesocosm experiments using *Skeletonema* strains [13] and other diatom species [23]. Irrespective of the inoculation, the phytoplankton composition was dominated by diatoms in every mesocosm. Depending on time and treatment, diatoms accounted for 70 to 94% of the total biomass in our experiments. In the Bothnian Sea diatoms dominate the spring bloom and were estimated to account for 50 to 70% of the total phytoplankton biomass [22]. The major diatom species found during a recent field survey were *Achnanthes taenicata*, *Chaetoceros* spp. and *Thalassiosira* spp. while *Skeletonema* spp. were present only in minor amounts [22]. This is also reflected in the uninoculated mesocosms CTRL and PUA+, where *Skeletonema* spp. reached only low cell counts. The second most abundant group of algae in all mesocosms was Dinophyceae, which is again in line with the phytoplankton composition of the Bothnian Sea [22]. Further, cell counts and chlorophyll a determinations confirmed that the mesocosms CTRL and PUA+ were suitable to test the effect of PUA on the plankton community in a close-to-field situation. SKE1 and SKE2 were ideally suited to test the potential effect of diatom blooms of very similar isolates from one species that have a different PUA production potential.

Recent work detected heptadienal and octadienal in concentrations of approximately 0.1 nM during a *S. marinoi* bloom in the Adriatic Sea [19]. In mesocosm studies, concentrations of approximately 1 nM PUA were detected during an induced *S. marinoi* bloom [18]. We thus selected initially an addition of PUA that was calculated to result in a final concentration of 1 nM. Nevertheless, only a fraction of this concentration was detected 23 h after PUA addition. This lower concentration was anticipated due to potential adsorption of PUA on the mesocosm containers, instability of the metabolites, microbial uptake and transformation, as well as potential loss due to volatility. However, after repeated PUA additions, concentrations up to 1 nM were reached. We can thus assume that up to day 6 the PUA addition resulted in concentrations that match roughly those found in nature. In our experiments, we added PUA as dilute aqueous solution and immediately mixed the mesocosms to reach a homogeneous concentration of these aldehydes. Simple hydrodynamic considerations suggest that PUA-producing diatoms will build up a concentration gradient of these metabolites around the cells, resulting in elevated local concentrations on a microscopic scale. We therefore also added 1000 nM heptadienal and 250 nM octadienal to overcompensate such potential local effects. Again, these concentrations were not detected after the first addition, but up to *ca.* 600 nM PUA were found after the repeated additions. Since we inoculated the SKE1 and SKE2 mesocosms with *S. marinoi *strains that were different in their PUA production, we could also test the effect of PUA release by otherwise very similar algae from one species. Indeed, PUA concentrations in SKE1 and SKE2 developed differently with SKE2 reaching higher PUA concentrations over the entire experiment. 

The bacterial abundance in all treatments was 1 × 10^6^ cells·mL^−1^ at the beginning of the experiment. Due to the filling procedure of the mesocosms, we conclude that microorganisms at day 0 consisted of the local bacterial community in the Bothnian Sea. During the first 10 days, no difference in the bacterial cell number between the 4 treatments were detected, indicating no effect of the addition of low amounts of PUA or the onset of *S. marinoi* blooms. Afterwards, both CTRL and PUA+ exhibited a significantly enhanced bacterial growth compared to SKE1 and SKE2. The suppression of bacterial growth in the two SKE treatments might result from a competition for essential nutrients [24], or from weak antibacterial activity of the algae. Such antimicrobial activity of *Skeletonema costatum*, recently renamed to *S. marinoi* [25], was lately observed. This alga produced antimicrobial substances particularly during the steady-state growth phase [26] that could explain the observed trend in bacterial abundance (Figure 3A). We can conclude, however, that this effect is not due to the PUA of *S. marinoi*, since comparable concentrations in the first days of the PUA+ treatment had no effect on the bacterial abundance, and actual measurements of bacterial production showed highest values in PUA+ (not shown). Since CTRL and PUA+ did not differ throughout the experiment, the addition of PUA has obviously no effect on bacterial abundance, even if concentrations way above those reached in nature were applied. Apparently, the antibacterial activity of PUA detected in laboratory studies [3,17] is not sufficient to inhibit bacterial growth in the plankton. This might be explained by the fact that in laboratory studies concentrations higher than 3 µM PUA are required to trigger activity, but the highest PUA concentration reached in the PUA+ mesocosms was only around 700 nM (Figure 4). It might, however, not be excluded that elevated concentrations of dissolved organic matter (DOC), resulting from the PUA addition, support bacterial growth. Such a positive effect might be counterbalanced by antibacterial activity of PUA. The virus abundance was also not affected by PUA. Similar to the trend observed for bacterial abundance, PUA+ exhibited significantly higher viral particle counts than SKE treatments, while not differing from the control at the end of the experiment. Thus, neither the added PUA nor the PUA released by *S. marinoi* negatively affects virus abundance in natural plankton assemblages or artificially induced diatom blooms. Since we did not see any increase of viral abundance during the decline of the *S. marinoi* bloom, we conclude that the diatom succession was not controlled by viruses. However, viral termination of phytoplankton blooms has been reported for the Prymnesiophyceae *Emiliania huxleyi* (e.g., [27,28,29]); also, diatom lysis by viruses has been observed [30]. However, since we did not separate between viruses infecting bacteria or phytoplankton, the more abundant bacteria viruses could mask changes in phytovirus abundances. 

Bacterial community composition is as important as bacterial abundance for the structure and succession of plankton communities. The bacterial community composition in all mesocosms was dependent on the growth stage indicated by a distinct grouping between samples taken during the beginning and towards the end of the experiment. Since PUA induce different effects on marine bacteria from different taxonomical groups in cultures [31], we also addressed the question of whether PUA or PUA-producing algae influenced the bacterial community composition in a close-to-field mesocosm environment. Using T-RFLP, we could not detect an effect of PUA addition on bacterial diversity. During the first PUA addition (day 3), in low concentrations equivalent to those detected in nature, we did not see any grouping within the cluster analysis. The CTRL, which was the only treatment without any detectable PUA at this time, was not separated from the two SKE treatments or from PUA+ in which subnanomolar concentrations of dissolved PUA were detected. Even the second PUA addition resulting in above-natural conditions did not cause detectable changes. Recent investigations have evaluated the effect of PUA addition on bacteria representing a natural community from the coastal area of the Mediterranean Sea [20]. Using nanomolar additions of PUA, no strong effect of PUA on the total abundance or the actively respiring cells was observed in short-term incubations. However, a decreased metabolic activity in response to PUA addition was detected, indicating that PUA may have the potential to shape the bacterial community composition. In our experiments, we compensated for potential limitations of short term incubations and tested the effect of different PUA concentrations over 16 days and found no detectable effect on bacterial diversity. This suggests that the postulated effects might be very subtle and cannot be observed even during an extended mesocosm experiment. Nevertheless, our methods are clearly suitable to pick up changes in the bacterial community, which is supported by the observation that bacterial communities differ in all mesocosms between early and late stages of the experiment (Figure 5B). The primers used in our PCR reaction can potentially amplify 16S rRNA of diatom chloroplasts. However, bacteria are in higher abundance than diatoms and the sequence similarity of the primers in comparison to *Skeletonema* chloroplasts is not high enough to ensure sufficient amplification. Thus, the influence of amplified eukaryotic rRNA can be neglected. This is additionally supported by the fact that we do not observe a differentiation between inoculated and non-inoculated treatments in the T-RFLP profiles which would be expected for amplified rRNA from *Skeletonema *chloroplasts. We saw a very distinct separation of the bacterial community in SKE2 after day 14. This change can most likely not be attributed to PUA since SKE1 was also releasing PUA at the same time, although in lower concentrations. Comparable specific shifts in bacterial communities were reported by several authors for diatom cultures. Laboratory studies revealed that unique bacterial communities develop if axenic cultures of the diatoms *Thalassiosira rotula* and *S. costatum* are inoculated with natural bacterial assemblages [32]. Other mesocosm studies with the diatom *S. costatum* demonstrated a change of the bacterial composition over time, similar to the results presented here [23]. The observed exclusive bacterial community in SKE2 at day 14 was indicated by a low similarity in the cluster analysis. However, three days later, the bacterial community of SKE2 grouped together with SKE1 indicating no long-lasting effect. Such dynamic temporal shifts in bacterial composition were shown already in mesocosm experiments [23] as well as in field studies [33]. Interestingly, complex patterns of metabolite release were shown recently for diatom species [34], which turned out to be crucial for the growth of competing phytoplankton species [35]. Thus, exudates of diatoms might influence the plankton community, but according to our study, the metabolites influencing bacterial communities are not PUA. 

## 4. Experimental Section

### 4.1. Experimental Design

The experiment was performed at the mesocosm facility of the Umeå Marine Science Center (UMSC, Umeå, Sweden), between 3 May (day 0) to 22 May (day 19) 2010. The experiment was run in 12 indoor polyethylene mesocosm towers of 5 m depth and a volume of approximately 2000 L each. Thermal advection, achieved by heating the lowest section of the tanks, resulted in a slow mixing of the water column with a turnover time of approximately 24 h. All tanks were filled with unfiltered seawater (29 April) with its natural microbial community from the Bothnian Sea and were kept at 8 °C to 10 °C, corresponding to ambient field conditions outside the mesocosm [36]. Nutrients were added to all tanks directly after filling to a concentration of 13 µM NaNO_3_-N, 4 µM NH_4_Cl-N and 4 µM NaH_2_PO_4_. The nutrient levels mimicked the unlimited conditions at the time of the spring bloom in the Bothnian Sea [36]. Nutrient levels were regularly monitored on days 8 and 14 and adjusted if necessary with nitrate and phosphate and additionally, on day 17, with silicate, to initial conditions. Four days after filling (3 May), we started 4 different treatments, each in triplicates. Two treatments were supplemented with two different *S. marinoi* strains (*S. marinoi* abbreviated SKE1 and *S. marinoi* abbreviated SKE2) having different growth and PUA-releasing characteristics. The addition of *Skeletonema* was adjusted to roughly double the concentration of chlorophyll *a* in the tanks after 4 days of growth and thereby leading to a *Skeletonema* dominated phytoplankton community throughout the experiment. Additionally, one treatment consisted of unfiltered natural seawater only (CTRL) and the final treatment consisted of unfiltered natural seawater that was supplemented with different concentrations of PUA at two different time points (PUA+). During days 1 to 4 we added daily hepta- and octadienal, corresponding to a final concentration of 1 nM, representing similar concentrations as found in *S. marinoi *dominated mesocosms [18]. Therefore, heptadienal (22 µL) and octadienal (24 µL) stock solutions (10 mg·mL^−1^ in methanol) were dissolved in 5 L 0.2 µm filtered seawater and thoroughly mixed. This 5 L seawater solution was afterwards added to each tank of PUA treatment and 46 µL of methanol in 5 L filtered seawater were added to the control tanks. All 12 mesocosms were subsequently mixed 3 times with a Secchi disk. To evaluate the effect of PUA on the natural microbial community under artificially elevated PUA concentration, we daily added heptadienal to a calculated concentration of 1000 nM and octadienal to a concentration of 250 nM during day 10 to day 13 of the experiment. Here 2200 µL of a heptadienal and 620 µL of an octadienal stock solution (100 mg·mL^−1^ in methanol) were added daily to 5 L of filtered seawater and which were handled identically to the low PUA supplement (addition of 2820 µL of methanol in 5 L seawater to the control). The relative PUA concentrations were adjusted to match the heptadienal/octadienal ratios produced by *S. marinoi *laboratory batch cultures [16]. All treatments were run for 21 days.

### 4.2. General Sampling

Samples for bacterial diversity, bacterial abundance, viral abundance, chlorophyll a, phytoplankton cell abundance and inorganic nutrient determinations were taken 7 to 8 times during the experiment depending on the analysis. In order to accomplish this, 10 L were sampled from each tank at a depth of approximately 1 m into a plastic container. All samples for the corresponding analysis were taken from this container. Samples (1 L) for dissolved PUA analysis were additionally taken daily at days when no regular sampling was scheduled to ensure complete PUA monitoring during the experiment. After each major sampling, all tanks we refilled with 0.2 µm filtered seawater to keep the water volume constant. 

### 4.3. Chlorophyll a Concentration

All samples were treated in duplicate under reduced light. Depending on cell abundance 100 or 50 mL portions of the sample were filtered through a 25 mm GF/F filter (Whatman, Kent, UK), using a vacuum of about 20 kPa. The filters were then transferred into 15 mL plastic Falcon tubes containing 4 steel balls of 5 mm diameter and blanks were prepared using Milli-Q water. A 10 mL portion of 95% ethanol was added to each tube, the stopper tightened firmly and a rack positioned with the tubes horizontally on a large amplitude linear shaker at about 200 rpm for 5 min, thereby crushing the filters thoroughly. Next, the tubes were kept overnight in the dark and the next day each tube was briefly shaken by hand prior to centrifugation at 3500 rpm for 10 min using a Heraeus Instruments Labofuge 400 (Hanau, Germany). The fluorescence of each solution was recorded on a Perkin Elmer LS 30 spectrofluorometer (Waltham, MA, USA) operating at excitation and emission wavelengths of 433 and 673 nm, respectively. The chlorophyll a content of each sample was calculated using a calibration constant obtained in a previous calibration.

### 4.4. Phytoplankton Abundance

Samples for phytoplankton identification and monitoring of phytoplankton abundance were fixed with 0.5% acid Lugol’s solution. Volumes of 10 or 3 mL of fixed samples were settled in a sedimentation chamber for 12 to 24 h and counted according to the Utermöhl technique [37] in an inverted microscope (NIKON Eclipse TE 300). Phytoplankton, identified to species or class level, were counted and measured for calculations of biomass using the equations and size classes described for the Baltic Sea [38].

### 4.5. Bacterial Abundance

Samples for total bacterial abundance including attached and free living bacteria (50 mL) were preserved with 0.2 µm filtered formaldehyde (1.5% final concentration). For analysis, 4 mL sample was filtered onto black 0.22 µm 25 mm polycarbonate filters (Osmonics Inc., Minnetonka, MN, USA) and stained with acridine orange dissolved in MilliQ water (0.01% final concentration). Samples were analyzed in an epifluorescence microscope (Zeiss Axiovert 100) connected to an image analysis system using blue excitation light and a 450 to 490 nm filter [39].

### 4.6. Viral Abundance

Virus samples (1 mL) were filtered subsequently through 0.8 and 0.2 µm Supor membrane syringe filters (Pall Corporation, Newquay, UK), fixed in glutaraldehyde (0.5% final concentration) for 30 min at 4 °C, frozen in liquid nitrogen, and stored at −70 °C [40]. The staining procedure with SYBR Green I (Molecular Probes Inc. Eugene, OR, USA) was modified from Marie *et al.* [40]. In short, virus samples were thawed at 35 °C for a few min and then diluted 100 times in 0.99 mL TE buffer (10 mM Tris-HCl and 1 mM EDTA, pH 8.0) to avoid coincidence (two or more particles being present at the same time in the sensor zone) [40,41]. Diluted virus samples were stained with 10 µL SYBR Green I or 10 min in the dark at 80 °C. Final concentration of SYBR Green I was a 10^−4^ dilution of the commercial stock. Virus samples were analyzed using a FACSCalibur flow cytometer (Becton Dickinson, San Jose, CA, USA) equipped with a 15 mW 488 nm air-cooled argon-ion laser and standard filters. The trigger was set to green fluorescence. Readings were collected in logarithmic mode and analyzed with the software CellQuest (version 6.0; Becton Dickinson, San Jose, CA, USA, 2007). A total of at least 5000 events were recorded for each sample. Green fluorescence (GFL), side scatter (SSC) and total events were recorded. The data were normalized to fluorescent beads (yellow beads of 1.5 µm; Polysciences Inc., Warrington, PA, USA) which were added as an external standard.

### 4.7. PUA Analysis

Sampling and analysis of dissolved PUA measurements were based on a protocol published previously [16]. Briefly, a 1 L sample was transferred to a 1 L glass bottle and 5 µL of a benzaldehyde solution (1 mM in methanol) were added as internal standard. First, this sample was transferred by vacuum through a sand cartridge to gently remove diatom cells. The filtrate was immediately loaded on a 3 mL EASY Chromabond cartridge (200 mg, Macherey-Nagel; Düren, Germany) previously treated with 1 mL of a 25 mM *O*-(2,3,4,5,6-pentafluorobenzyl)hydroxylamine hydrochloride (PFBHA) solution in 100 mM TRIS-HCL at pH 7.2. The EASY cartridge was rinsed with water and dried by applying a gentle vacuum. PUA were eluted with 4 mL of a 5 mM PFBHA solution in methanol under gravity and stored at −80 °C until further extraction. Extraction was performed in 25 mL glass flasks filled with 8 mL hexane and 8 mL water. After addition of 1.5 mL of concentrated sulfuric acid (95%), the hexane phase was separated and dried with sodium sulfate. The hexane phase was then evaporated to dryness under reduced pressure and the remaining derivatized PUA were re-dissolved in 100 µL hexane. GC-MS measurements were performed on an Agilent 6890N gas chromatograph equipped with a DB-5ms 30 m column (0.25 mm internal diameter, 0.25 µm film thickness) coupled to a Waters GCT Premier mass spectrometer (Manchester, UK). Helium was used as carrier gas at a constant flow rate of 1 mL·min^−1^. The calibration was performed using synthetic standards of heptadienal and octadienal normalized to benzaldehyde as internal standard with *R*^2^ = 0.9794 and *R*^2^ = 0.9764 for heptadienal and octadienal, respectively.

### 4.8. Bacterial Community Composition

For DNA extraction, 100 mL of sample was filtered on 47 mm diameter, 0.22 µm pore size polycarbonate filters (GE Water & Process Technologies, Fairfield, CT, USA). Filters were stored at −80 °C until DNA extraction which was carried out as described previously [42]. For T-RFLP analysis the 16S rRNA-gene was partially amplified by PCR using bacterial 16S primers 27F_FAM (AGA GTT TGA TCC TGG CTC AG) [43,44] and 926R_HEX (CCG TCA ATT CCT TTG AGT) [43]. The reaction mixture consisted of 1 µL of non-diluted, half-diluted or 10^−1^ diluted template DNA, 25 mM dNTPs, 10 µM of each primer, 10× buffer and 0.5 units of KAPA Taq polymerase (KAPA Biosystems, Woburn, MA, USA) for 25 µL reaction. The amount of PCR products were estimated in a 1% agarose gel and then purified with a PCR purification kit (Macherey-Nagel, Düren, Germany). Restriction of purified PCR products was done with HhaI and RsaI restriction enzymes separately. After a restriction time of 6 h, products were precipitated in ethanol solution (the final mixture containing 97% ethanol with 5 v% 3 M NaOAc) for 1 h at −70 °C. Then, samples were centrifuged in a microcentrifuge for 30 min at 15,000 rpm and supernatants were carefully removed. Pellets were washed with 500 µL 70% ethanol and centrifuged for 10 min at 15,000 rpm. Finally, samples were left to air-dry in a laminar hood and re-suspended in 10 µL of sterile water. For the T-RFLP run with an ABI analyzer, 2–6 µL of sample (depending on the amount of PCR product) was mixed with 6–10 µL of Hi-Di solution (Applied Biosystems, Carlsbad, CA, USA) and 0.01 µL of size standard 600LIZ (Applied Biosystems). Each sample was measured twice and both technical replicates were used for the analysis. The fluorescent peak profiles were analyzed with Peak Scanner software (Applied Biosystems, Carlsbad, CA, USA); the peak data was processed and the peak profiles constructed using T-REX [45], a web-based open access program. The T-REX uses the approach of Abdo *et al.* [46] for noise filtering and the method of Smith *et al.* [44] for peak alignment. The program default values were used for noise filtering, peak alignment was done using a clustering threshold of 0.6 and relative peak heights were used for constructing the peak profiles. The community profiles were clustered based on the Bray-Curtis similarity matrix of log(x + 1) transformed data using PRIMER-6 (version 6.1.12; Primer-E Ltd., Plymouth, UK). Because of the high number of peaks in each profile (due to two fluorescent primers) and because we used peak heights for calculation of results, the maximum similarities between any samples were restricted to approximately 97%. However, based on 75% similarity observed between some of the replicates from the same tanks, we considered that sample profiles that had more than 70% similarity represented similar communities. Samples with replicates less similar than this were removed from the analysis. The evaluated peak heights reflect the relative amounts of different species in the sample and thus give more information on the bacterial community structure than presence/absence data. However, they add to the total complexity of the community profiles, thus reducing the similarity between samples.

### 4.9. Statistical Analysis

Difference in cell abundance and chlorophyll a data were assessed using a Repeated Measures Analysis of Variance (RM-ANOVA) with the Tukey *post-hoc* test. The analyses were performed with the software package Sigmaplot 11 (Systat Software Inc., San Jose, CA, USA, 2008).

## 5. Conclusion

In summary, we show that PUA are released by *S. marinoi* and that these aldehydes did not affect the abundance of both bacteria and viruses. Additionally, PUA did not affect the natural bacterial community composition under environmentally realistic and also not at elevated concentrations (1000 fold). Moreover, we observed that different *S. marinoi* strains have unique bacterial communities which change in a dynamic way. Taken together, these results suggest that PUA play no significant role in diatom-bacteria interactions but do not exclude an influence on interactions with other co-existing organisms.
